# Supplementary material for: Outcomes of Importance to Patients in Reproductive Genetic Carrier Screening: A Qualitative Study to Inform a Core Outcome Set
Source: J Pers Med. 2022 Aug 12;12(8):1310. doi: 10.3390/jpm12081310 (PMC9409855; doi:10.3390/jpm12081310)
Supplement: Supplementary file 1 [file jpm-12-01310-s001.zip › jpm-1786560-supplementary.pdf]

DESCRIPTION OF SUPPLEMENTAL DATA

Supplementary Table S1 – Interview schedule

Supplementary Table S2 – Outcome domains with definitions and examples (new domains or updated domains indicated in blue)

Supplementary Table S1: Example Search Strategy (Medline Ovid)

Welcome, introductions and explanation	
**5 minutes**	**Welcome**<br>Introduce moderator and note taker.<br><br>**Pre-amble:**<br>Thank you for speaking with me today, I appreciate you taking the time.<br>I'm a genetic counsellor currently completing a PhD and this interview is part of an overarching study looking at the impact of carrier screening on patients such as yourself who accessed this testing. As researchers, we do this by defining what we call 'outcomes', which is essentially something measurable that captures an aspect of your experience. I'll give you a detailed example of an outcome later in the interview. Our goal is to define 5-10 outcomes that when looked at all together give a good overall picture of your experience of carrier screening, which can then guide what researchers measure in the future.<br><br>To start, I'm going to ask you to answer some broad questions that relate to your experience of carrier screening and then do an exercise where you think of some words that capture what it was like for you to have carrier screening. From this discussion together we'll come up with outcomes that relate to the words you use to describe your experience.<br><br>This interview will last for up to an hour. I'll be video and audio recording the interview for our analysis and will store the recording securely for use in future research. If at any time you would prefer not to answer a question that is fine. If you want to take a break or do not feel comfortable, please let me know. Our discussion will be strictly confidential and you will not be identified in our results. We have ethics approval for this study.<br><br>Did you have any questions for me before I start the recording [*if yes, address question]*.<br><br>[*start recording*] Thank you once again for agreeing to participate. I'll get you to indicate your verbal consent and we'll start.
**Part 1: Exploratory Questions**	
**10 minutes**	**Firstly, I need to ask for some basic demographic information which we use to give some context around your answers:**<br>1. How old are you?<br>2. What is your highest level of education?<br>3. What is your ethnic background?<br>4. Do you have any family history of a genetic condition?

	**Can you tell me how you found out about carrier screening and why you decided to have this test?**<br>Prompts [*if needed*]:<br>1. Tell me about your results?<br>2. How did you feel throughout the process?<br>3. Is there anything you felt was particularly good or bad about having carrier screening?
**Part 2: Adapted Nominal Group Technique – Word Association Exercise**	
**20 minutes**	The rest of the session focuses on being interactive and collaborative. We are going to do a word association exercise, and what I want you to do is keep the experience of carrier screening that you've just discussed at the forefront of your mind. I'm going to break up the process of carrier screening into four time periods for us to think about:<br>1. Before testing<br>2. Waiting for your results<br>3. Receiving your results and the immediate time following that<br>4. Now, looking back on the experience so far and summing it up as a whole<br>I'm going to prompt you to think about what was happening during each of these time periods and get you to take a few moments to write down some words that come to mind to sum it up. We'll then use the words that you come up with to decide on an outcome that they relate to.<br>You can write down single words, or a short string of words, and you can write down as many as you feel like.
	**1|** Firstly, think about the time leading up to carrier screening, when you spoke to your health care provider and decided to have this test. You might want to think about what information you were given, what your motivations were, and how you were feeling at that time. Write down some words that come to mind.
	**2|** Next, think about the period of time while you were waiting for your results, what was that like for you, how were you feeling, and write down some more words.
	**3|** Next, think about when you got your results. Think about the information you were given, how were you feeling, think about what those results meant for you and what you had to do next. Pick a few words that sum up that time.
	**4|** Lastly, think about your perspective now, the bigger picture as you are looking back at the experience. Write down a few words that sum up your feelings overall about carrier screening and the impact it has had on you.

<table>
<tr><td></td><td>Prompts [if participant needs help thinking of words]:
How did the testing impact on you personally, on you and your partner as a couple, or your family more broadly?
How did you feel throughout the process?
What did you understand about the test?
How did the timing of testing work for you?
How supported did you feel?
How worthwhile was the test and why?
What were the benefits and/or harms, if any?</td></tr>
<tr><th colspan="2">Break</th></tr>
<tr><td>5 minutes</td><td>We have time for a short break now if you’d like [check if they would like a break] If yes, please don’t leave the meeting but feel free to turn your cameras and microphones off and come back online in 5 minutes [tell them what time to come back]</td></tr>
<tr><th colspan="2">Part 3: Adapted Nominal Group Technique – Eliciting Outcomes</th></tr>
<tr><td>20 minutes</td><td>Now I’m going to use this online whiteboard to write down the words that you’ve chosen and then we’ll discuss each of them to get some context around the word and think about a research outcome that it relates to. Before we start, let me give you an example of what we mean by ‘outcomes’. Take COVID-19 as an example. If a researcher wanted to find out what impact the pandemic has had on people, they could look at a variety of different outcomes, they might look at:
People’s mental health by measuring levels of depression, in that case the outcome being looked at would be depression. You could also look at different mental health outcomes like anxiety for example.
Depression
Anxiety
How people changed their behaviour, like wearing a mask.
Uptake of wearing a mask in public
How informed people felt. One way to measure this would be to ask if people were satisfied with the information that was available about COVID; satisfaction with information provision. Or you could measure their understanding, by asking questions about COVID and seeing how many they get right; Knowledge about COVID.
Satisfaction with information provision
Knowledge about COVID
If you were to look at all of these outcomes across a number of people, you would start to capture a broad picture of the experience of the pandemic, and this is what we want to do for carrier screening. Does that make sense? Any questions?</td></tr>
</table>

	Let's look at the first time-frame. Can you tell me what words you wrote down when thinking about the time before you had testing and when you were deciding to have it [*write down words on the whiteboard*]
	Can you give me a bit of context around this word? *[through discussion, associate the word with an outcome. This is a collaborative process with the participant so listen to the words they are using. Consider the surface layer of meaning within their explanations and prompt them to explore more underlying or nuanced meanings that you perceive during the discussion.]*<br>[*Repeat for all words*]
	Do you think that this outcome that I've written down captures your meaning? *[try to capture outcomes that closely reflect the participant's own words. Check-in with the participant and adjust the wording until they are happy that it reflects their intended meaning]*<br>[*repeat for all time-frames*]<br>• Tell me what words you wrote down when thinking about waiting for your results<br>• Tell me what words you wrote down when thinking about receiving your results and the immediate time afterwards<br>• *[if two-step screening]* Tell me what words you wrote down when thinking about waiting for your partners results<br>• *[if two-step screening]* Tell me what words you wrote down when thinking about receiving for your partners results<br>• Tell me what words you wrote down when thinking from your perspective now, some words that sum up your feelings about carrier screening now looking back on the experience
**Part 4: Adapted Nominal Group Technique – Prioritising Outcomes**	
	Now we are going to consider all the outcomes we have and think about which of these you think would be the most important for researchers to capture from your perspective as someone who has had reproductive carrier screening. Try to pick three that you think are the most important and consider why you made that choice. Take a few moments now to pick your top 3.<br><br>Can you tell me what your number 1 most important outcome was and why?<br>*[repeat with second and third ranked outcomes]*<br><br>Prompts:<br>• Why do you think you ranked [outcome] high?
**Summary & Conclusion**	
**5 minutes**	Provide an overall summary of the session:<br>1. Do you feel that that is an adequate summary of what we discussed?<br>2. Have I missed anything or is there anything else anyone would like to add?<br>3. I'd like to ask for your feedback on the experience on this interview and if you have any specific feedback regarding the word association exercise?<br>If you know anyone who would be interested, please pass on the details of the survey [*send f/u email with link to survey if they want to snowball*]<br>Inform about Delphi and obtain to consent to contact them to participate. Thank them for their time and close the meeting.

Supplementary Table S2: COMET/CODECS Taxonomy

Core Area	Outcome Domain (COMET taxonomy)	Sub-domain (defined by SMG)	Definition	Example outcome
Physiological / clinical	Congenital, familial, and genetic outcomes	Primary laboratory outcomes	Outcomes related to the core findings or results reported in RGCS	*- Carrier detection rate/carrier status of participants*<br>*- Identification of increased risk couples*
		Secondary or incidental laboratory outcomes	Outcomes related to laboratory findings not related to the primary indication for screening.	*- Identification of variants of uncertain significance*<br>*- Identification of homozygous, hemizygous or compound heterozygous individuals at risk for developing one of the screened conditions*
		Technical laboratory outcomes	Outcomes related to technical or practical considerations of RGCS from the laboratory perspective	*- Rate of test failure due to insufficient DNA in patient sample*<br>*- Rate of laboratory error (false negatives or false positives)*
	Pregnancy, puerperium and perinatal outcomes	Pregnancy outcomes	Outcomes related to the impact of screening results on pregnancy outcomes.	*- Results of prenatal diagnosis*<br>*- Decision to continue or terminate affected foetuses in future pregnancies*<br>*- Number of individuals born with the condition(s) being screened for*
Life Impact	Cognitive functioning	Patient attitudes, perceptions and beliefs related to RGCS	Outcomes related to patient's attitudes, perceptions or beliefs about RGCS	*- Perception that RGCS would alter reproductive decisions*<br>*- Perceived chance of a carrier finding*
		Deliberation and informed choice	Outcomes related to making an informed choice to undertake RGCS	*- Deliberation on the decision to accept or decline testing*<br>*- Informed choice (congruence of attitudes, knowledge, and test uptake)*
		Knowledge and understanding	Outcomes related to knowledge, incorporating concepts of understanding, recall and retention.	*- Knowledge before and after pre-test genetic counselling*<br>*- Recall of correct screening result at a later timepoint*
	Delivery of care	Uptake of services	Outcomes related to actual or intention to uptake an offer of RGCS	*- Uptake of RGCS*<br>*- Intention to accept the offer of RGCS*
		Barriers, facilitators and factors influencing patient experience	Outcomes related to reasons for and against uptake of services, including offers of RGCS and further testing, as well as factors that influence experience of these services	*- Reasons for accepting/declining an offer of RGCS*<br>*- Reasons or factors related to emotional reactions and psychological wellbeing*<br>*- Sources of additional information used for decision-making regarding uptake of RGCS*

		Genetic counselling resource use	Outcomes related to the use and conduct of genetic counselling services	*- Number of post-test genetic counselling consultations*<br>*- Time required for pre-test genetic counselling session*
		Goals of pre- and post-test genetic counselling	Outcomes related to the patient experience of pre- and post-test interactions with their health providers, and whether needs are met by their providers	*- Genetic counselling supported informed decision-making*<br>*- Timing and method of information provision promoted understanding*
		Patient preferences	Outcomes related to patient preferences regarding the offer of RGCS	*- Preference regarding individual or couple-based results*<br>*- Preference regarding conditions included in RGCS*
		Patient satisfaction with the processes of RGCS	Outcomes related to patient satisfaction with services provided during RGCS	*- Satisfaction with pre-test genetic counselling*<br>*- Satisfaction with accessibility, cost and convenience of the screening process*
		Timeliness	Outcomes related to the timeliness of delivery of care in RGCS programs	*- Mean gestational age at time of reproductive carrier screening*<br>*- Offer of reproductive carrier screening to women before 10 weeks gestation*
	Emotional functioning/wellbeing	Decision satisfaction and regret	Outcomes related to decisional satisfaction or regret at a later timepoint	*- Retrospective satisfaction with the decision to have RGCS*<br>*- Regret related to reproductive decision-making at a later timepoint*
		Psychological wellbeing	Outcomes related to the psychological impact of RGCS	*- Anxiety (measured at a range of timepoints)*<br>*- Grief and loss (perception of pregnancy journey and expected future)*
	Perceived health status	Perception of personal health status after RGCS	Outcomes related to the impact of RGCS on perception of personal health	*- Impact of results on perception of own health*
	Personal circumstances	Decision-making (non-reproductive)	Outcomes related to the impact of results on decisions other than reproductive planning	*- Decisions regarding long-term care, disability, or life insurance*<br>*- Lifestyle changes*
		Decision-making (reproductive)	Outcomes related to impact of results on decision-making for reproductive planning, including perceived or actual impact on these decisions	*- Pursued or planned to pursue alternate reproductive options*<br>*- Intended reproductive decisions if identified as an increased risk couple*
		Familial implications	Outcomes related to the impact of results of patient relationships	*- Impact of results on couple's relationship*<br>*- Number of heterozygotes that informed family members of their results*

		Perceived utility of RGCS	Outcomes related to patient's perceptions of the impact of RGCS and how they utilised the results	- *Confidence or empowerment related to reproductive decision-making*<br>- *Results were available in a timely manner that allowed for consideration and decision-making*
	Social functioning	Acceptability of further testing or alternative reproductive options	Outcomes related to patients' perspectives, and wider societal perspectives, on prenatal diagnosis, termination of pregnancy, and preimplantation genetic diagnosis	- *Personal preferences regarding PND, PGD and TOP*<br>- *Perception of societal acceptability of PND, PGD and TOP*
		Privacy concerns and stigmatisation	Outcomes related to the impact of results on privacy and stigmatisation	- *Concern regarding privacy or confidentiality*<br>- *Concern regarding insurance*
Resource Use	Need for further intervention	Further testing	Outcomes related to the use of further testing for various purposes including clarifying reproductive risk as a couple, testing during a pregnancy, or electing PGD	- *Uptake of partner testing*<br>-*Uptake of prenatal diagnosis in increased risk pregnancies at the time of screening*<br>- *Uptake of preimplantation genetic diagnosis in increased risk couples*
